# A Preventive Behavior Questionnaire for Older Workers in Brazil: Protocol for a Cross-Cultural Adaptation and Validation Study

**DOI:** 10.2196/95636

**Published:** 2026-09-15

**Authors:** Ihana Thais Guerra de Oliveira Gondim, Ana Karina Pessoa da Silva Cabral, Maria das Graças Wanderley de Sales Coriolano, Alexandra Lecours

**Affiliations:** 1 Département d’ergothérapie Université du Québec à Trois-Rivières Trois-Rivières, QC Canada; 2 Programa de Pós-Graduação em Gerontologia Universidade Federal de Pernambuco Recife, Pernambuco Brazil

**Keywords:** occupational health, health behavior, safety management, surveys and questionnaires, psychometrics, validation study, cross-cultural comparison, transcultural studies, workers, aged, Brazil, preventive health services

## Abstract

**Background:**

Population aging is rapidly reshaping the Brazilian labor force, increasing the participation of adults aged 50 years and older and intensifying the need for safe, inclusive, and age‑friendly workplaces. Preventive behaviors at work play a vital role in protecting workers’ physical, social, and psychological health; however, no validated Brazilian Portuguese instrument is currently available to measure the frequency of these behaviors. The Preventive Behaviors at Work Frequency Scale (Échelle de Fréquence des Comportements Préventifs au Travail), developed in Canada, evaluates how often workers adopt 6 key preventive behaviors grounded in the model of preventive behaviors at work. A culturally adapted and psychometrically validated version is therefore needed to support occupational health research, surveillance, and evidence-based interventions for Brazilian older workers.

**Objective:**

This study aims to describe the protocol for the cross-cultural adaptation and psychometric validation of the Brazilian Portuguese version of the Preventive Behaviors at Work Frequency Scale for workers aged 50 years and older.

**Methods:**

This methodological study will be conducted based on international guidelines for cross-cultural adaptation and questionnaire validation and will be completed in six steps: (1) forward translation, (2) forward translation synthesis, (3) back translation, (4) harmonization and expert appraisal of relevance, (5) pretesting with the target population, and (6) field-testing and psychometric evaluation. Data collection will be conducted over a 12-month period among Brazilian workers aged 50 years and older.

**Results:**

A Mitacs grant was obtained for 2025 to 2026. Translation, cross-cultural adaptation, and pretesting involving 30 participants were completed between February 2026 and August 2026. Field-testing and psychometric evaluation are scheduled to begin in September 2026, with data collection and analysis expected to be completed by the end of February 2027. Study findings are anticipated to be published in 2027.

**Conclusions:**

This study will provide the first Brazilian Portuguese version of the Preventive Behaviors at Work Frequency Scale for workers aged 50 years and older. The adapted instrument is expected to support research and evidence-based workplace interventions aimed at promoting healthy and sustainable aging at work. Furthermore, the methodological framework described in this protocol may guide future cross-cultural adaptation and psychometric validation studies.

**International Registered Report Identifier (IRRID):**

DERR1-10.2196/95636

## Introduction

### Background

The global trend of population aging, combined with Brazil’s socioeconomic dynamics, has significantly reshaped Brazil’s labor force, thereby increasing the presence of individuals aged 50 years and older and making inclusion strategies a structural requirement for economic and social stability [1,2]. Worldwide, the number of people aged 60 years and older is projected to rise from 1.1 billion in 2023 to 1.4 billion by 2030 [3], underscoring the need for age-friendly work environments and policies to combat ageism. In the Brazilian context, these demographic shifts are evidenced by the proportion of individuals aged 60 years and older (15.6% of the total population), which has already surpassed the share of youth aged 15 to 24 years (14.8%) since 2023. The average age of the Brazilian population increased from 28.3 years in 2000 to 35.5 years in 2023 and is projected to reach 48.4 years by 2070 [2]. These demographic changes not only reshape population structures but also pose concrete challenges for the labor market, particularly regarding the inclusion of older workers.

Individuals aged 50 years and older must be integrated into the labor market under safe and adequate conditions. This group represents approximately 18% of formal employment in Brazil, totaling approximately 8.3 million workers [4,5]. Despite this substantial presence in the formal sector, informality (ie, employment without formal registration, a written contract, or access to social security contributions and labor rights) remains disproportionately high: rates reach 40.6% among workers aged 50 to 59 years and rise to 53.8% among those aged 60 years and older [4]. However, employment opportunities available to these workers in both formal and informal sectors are frequently characterized by lower wages and reduced job quality [4].

This demographic’s continued participation in the workforce is influenced by factors such as the increase in the minimum retirement age under recent social security legislation in Brazil; financial considerations, including supplementing retirement income or contributing to household earnings; and the desire to remain active and socially integrated through work, which is often closely linked to an individual’s social identity [4,6,7]. Nevertheless, remaining in the workforce is not without consequences as older workers face specific risks that demand attention.

Beyond adverse employment conditions, occupational health is another critical dimension as integrating this population into the workforce entails unique challenges such as an increased risk of disability following an occupational injury and a more complicated recovery period [8,9]. In the Brazilian context, 744,477 occupational accidents were recorded in 2024, and 133,583 involved individuals aged 50 years and older, accounting for approximately 18% of all leaves due to occupational injury [10]. Although this group accounts for a smaller proportion of cases, it consumes disproportionately more benefit time because recovery is typically longer, averaging 45 to 60 days compared to the overall population mean of 38 days [10]. These extended recovery periods are attributed to age-related structural and functional degeneration of tissues and bones, as well as comorbidities that impair healing and increase the complexity of rehabilitation [8,9]. Occupational health issues represent a major global public health concern [11,12], affecting workers, families, and organizations by reducing work performance and productivity [13,14] and imposing significant economic costs through health care and social security expenditures [15].

According to the International Labour Organization, the global costs of workplace accidents and occupational illnesses amount to nearly US $3 trillion, representing approximately 3.94% of the world’s gross domestic product [12]. The Brazilian Ministry of Labor and Employment estimates that occupational accidents account for a loss of up to 4% of the country’s total economic output. The impact is so significant that, in 2025, a national campaign for the prevention of occupational accidents aimed to raise awareness of occupational safety and health standards, fostering debate and encouraging the adoption of preventive measures to ensure safer working environments for Brazilian workers [16].

The prevention of occupational injuries and prolonged disability cannot be attributed solely to individual factors; rather, it reflects a dynamic interplay among personal, organizational, and systemic determinants, as conceptualized by the ecological model of disability management [17,18]. This model emphasizes that workplace practices, health care interventions, and legislative frameworks operate interdependently, making multilevel collaboration essential to prevent prolonged disability and ensure sustainable work participation [18]. Within this integrated system, workers play a crucial role at the individual level, particularly through preventive behaviors, which interact with organizational practices and broader systemic conditions [19,20]. The concept of preventive behaviors at work or related constructs has been explored in various fields of the literature, including psychology [21,22], work organization and management [23,24], ergonomics [25], and occupational therapy [20,26,27].

Psychological research typically conceptualizes preventive or safety behaviors at work within 2 primary dimensions: carefulness and safety initiatives [21,22]. The domain of work organization and management emphasizes information exchange, problem-solving, and employee involvement as central elements for driving organizational change [23,24]. From an ergonomic perspective, the analysis emphasizes the observation of work processes to identify risks and the worker’s ability to apply knowledge in strategic decision-making for prevention. Ergonomic literature further highlights contextual influences such as work intensity and duration, physical and mental workload, task division, interpersonal relationships, and biomechanical aspects [25].

Authors in the field of occupational therapy have defined preventive behaviors at work as observable and measurable actions that workers may adopt to protect their own health and safety as well as that of their colleagues, thereby contributing to overall occupational health. Importantly, these actions extend beyond the prevention of physical accidents to include social and mental health dimensions [26]. The literature indicates that these behaviors play a vital role in occupational health and safety performance [28], reinforcing the need for instruments capable of measuring them.

This definition of preventive behaviors is supported by the assumptions of the model of preventive behaviors at work (MPBW), which is based on the results of 3 studies combining data from scientific literature and empirical observations in the domains of physical safety, psychological health, and social health [29-32]. The MPBW describes 6 types of behaviors that workers can adopt to preserve their health at work in relation to the context in which they operate. These behaviors are (1) adopting a reflective practice; (2) pondering rules and procedures; (3) taking initiatives for health, safety, and well-being; (4) caring about others; (5) communicating; and (6) adopting a healthy lifestyle. The model also distinguishes the direction of these behaviors, which may be oriented toward the worker, colleagues, or the organization. It integrates contextual factors specific to the individual (eg, personal resources) and to the organization (eg, social support and workplace civility), which influence a worker’s ability to adopt these behaviors. Finally, the model posits that these behaviors can generate positive effects on indicators related to the person, the organization, and society, such as work motivation, reduction in occupational injuries, or the development of a culture of prevention. The model was subjected to different stages of content validation with various stakeholders [20,26,27].

On the basis of this model, researchers developed the Échelle de Fréquence des Comportements Préventifs au Travail (Preventive Behaviors at Work Frequency Scale; EFCPT), a French-language questionnaire that measures, for a typical work week, how frequently a worker adopts each of the 6 behaviors in the MPBW taking the work context into account. The EFCPT consists of 23 items rated on a 5-point Likert scale (1=never; 5=always), enabling calculation of dimension-specific scores to generate behavioral profiles or a total score for an overall view of adherence to preventive behaviors [33]. Psychometric analyses demonstrated a 6-factor structure explaining 64.75% of the variance, satisfactory internal consistency for each dimension (McDonald ω>0.70), and high test-retest reliability with intraclass correlation coefficients (ICCs) from 0.76 to 0.89, supporting the instrument’s validity for assessing preventive behaviors [33].

Validated Brazilian Portuguese versions of instruments addressing important dimensions of occupational health are available, such as the Job Stress Scale (demand-control-support) [34], Copenhagen Psychosocial Questionnaire I and II (psychosocial risks) [35-37], Work Role Functioning Questionnaire 2.0 (functional capacity) [38,39], Resilience at Work Scale (resilience) [40], and Healthy Work Environment Assessment Tool (healthy environment) [41]. More recently, the Brazilian version of the Centers for Disease Control and Prevention Worksite Health ScoreCard [42] was adapted and validated as a comprehensive tool for assessing organizational-level health promotion strategies. However, none of these instruments measures the frequency of preventive behaviors adopted by workers, which is the central focus of the EFCPT and a critical component for monitoring individual-level practices that sustain workplace health promotion. In older adults, aligning preventive behaviors into daily routines is essential for long-term maintenance as repetition fosters automatization, with behavior frequency serving both as evidence of consolidation and as a target for intervention, reinforced through self-assessment and awareness [33,43].

By introducing a behavioral perspective and identifying specific practices that promote health, safety, and well-being at work, the EFCPT addresses a critical gap in occupational health assessment. Validating this instrument in Brazil is essential to capture effective workplace preventive practices within the national context, supporting research, policy development, and interventions aimed at fostering healthier and safer work environments. Given the study’s focus on healthy and sustainable aging at work, the translation, cross-cultural adaptation, and psychometric evaluation of the EFCPT will be conducted specifically among workers aged 50 years and older. Cross-cultural validation will ensure linguistic and conceptual equivalence as well as reliability and comparability, enhancing the instrument’s applicability in this population.

### Aims

This paper aims to describe the study protocol that will be used to conduct the Brazilian Portuguese transcultural adaptation and validation of the EFCPT for workers aged 50 years and older.

## Methods

### Design

This study constitutes methodological research focusing on cross-cultural adaptation and psychometric validation. This type of study aims to translate, culturally adapt, and evaluate the psychometric properties of measurement instruments (scales or questionnaires) to ensure equivalence with the original version in semantic, conceptual, and cultural terms, as well as reliability and validity for the target population [44,45].

Cross-cultural adaptation and psychometric validation are inherently complex processes, and Cruchinho et al [46] emphasize that insufficient justification for methodological decisions and limited documentation are frequent sources of bias that undermine reproducibility. Our aim in drafting this protocol was to bring clarity, rigor, and traceability to all stages of the adaptation of the EFCPT into Brazilian Portuguese, thereby contributing to more standardized methodological practices and supporting the generation of robust evidence for workers aged 50 years and older.

### Original Instrument

The EFCPT [33] is a questionnaire originally developed in Canadian French to assess the frequency of preventive behaviors at work. The instrument comprises 23 items distributed across six dimensions: (1) adopting a reflexive practice (4 items); (2) pondering rules and procedures (3 items); (3) taking initiatives for health, safety, and well-being (4 items); (4) caring about others (4 items); (5) communicating (4 items); and (6) adopting a healthy lifestyle (4 items). Responses use a 5-point Likert scale (“never,” “rarely,” “sometimes,” “often,” and “always”), with an additional “not applicable” option when an item does not apply to the participant’s work context. Dimension scores and an overall score are calculated according to the original instrument guidelines. A score is calculated for each dimension by summing the scores of the corresponding items and dividing the total by the number of valid responses within that dimension, excluding items marked as “not applicable.” The overall score is calculated using the same procedure across all 23 items, dividing the sum of all valid item scores by the total number of valid responses. Higher scores indicate more frequent engagement in preventive work behaviors.

### Procedure and Analysis

This study is based on international guidelines for cross-cultural adaptation of measurement instruments [44-46] and comprises six sequential steps, which are (1) forward translation, (2) forward translation synthesis, (3) back translation, (4) harmonization and expert appraisal of relevance, (5) pretesting with the target population, and (6) field-testing and psychometric evaluation. The study commenced in February 2026 and is expected to be completed within 12 months.

#### Step 1: Forward Translation

Two independent translators (T1 and T2), both native Brazilian Portuguese speakers fluent in Canadian French, will translate the original French version of the instrument into Brazilian Portuguese [44-48]. One of the translators (T1) will be a health professional familiar with the instrument’s construct and content area, and the other will be a professional with no familiarity with the topic (T2), ensuring the capture of diverse linguistic interpretations [44-46]. During the translation process, both translators will record their doubts and comments using a form provided by the researchers [45,46]. Emphasis will be placed on conveying the intended meaning rather than adhering to literal translation [44,49].

#### Step 2: Forward Translation Synthesis

A committee approach will be adopted to synthesize the 2 previous translations into a single version [44-46]. This process will be conducted by the translators from step 1 (T1 and T2) and the principal researcher [45,46]. Prior to the consensus meeting, the principal researcher will prepare and distribute a comparative table containing the T1 and T2 translations, including the doubts and comments raised by each translator, along with the original version of the instrument [44,46]. During the meeting, the principal researcher will moderate the discussions and document the decisions [45,46]. The report for this stage will describe the consensual solutions adopted to address ambiguities and discrepancies and will be reviewed by the research team [46]. It is essential that all issues are resolved through consensus rather than individual compromise [45]. If necessary, the team will contact the instrument developers to clarify any remaining doubts regarding item translation [46].

#### Step 3: Back Translation

Another 2 independent translators (T3 and T4), both proficient in Canadian French and Brazilian Portuguese, will perform the back translation of the consensus forward version into Canadian French [46]. One translator (T3) will be familiar with health‑related constructs, whereas the other will not be familiar with the constructs (T4) [44,46], and neither will have prior access to the instrument (blinding) [44-46]. This stage will be conducted in a blinded manner for both translators to ensure its effectiveness in identifying conceptual discrepancies and ambiguities [46]. As a result, 2 versions in the original language of the instrument will be produced [46].

#### Step 4: Harmonization and Expert Appraisal of Relevance

##### Harmonization

A team-based approach will be used to compare the original instrument, the synthesized forward translation, and the 2 back translated versions to identify ambiguities, discrepancies, and cultural nuances and agree on the most appropriate translation [44-46]. This approach will involve a multi-professional committee [44-46,50] composed of 1 methodologist (the principal researcher) [44,45]; 4 Brazilian occupational health professionals who are familiar with the construct and content areas of the instrument [44,45]; all 4 bilingual translators involved in the translation and back translation (T1, T2, T3, and T4) [44-46]; 1 linguistic expert [45]; and the main developer of the original instrument [44], who will participate in a consultative capacity.

Prior to the harmonization meeting, the principal researcher will compare all versions (original, forward translations, synthesized version, and back translations) to identify issues for discussion [46,51]. During the meeting, the committee will review the instructions, each item, and the response format of the questionnaire using the 4 fundamental equivalences proposed by Beaton et al [45]:

- Semantic equivalence—accuracy of meaning, appropriate grammatical use, and conceptual precision
- Idiomatic equivalence—adequacy of cultural equivalents for colloquialisms or idiomatic expressions
- Experiential equivalence—confirmation that the experience described in the item occurs or is recognizable in the Brazilian context
- Conceptual equivalence—verification that the underlying concept holds the same cultural meaning (eg, work relationships and preventive practices)

The translators and the linguistic expert will contribute to semantic and idiomatic equivalence, whereas the occupational health professionals and researchers will ensure experiential and conceptual equivalence of the adapted version of the EFCPT. The committee members must discuss and reach a documented consensus on the instrument [44,46]. For partial or total replacement of items, researchers will need to obtain approval from the developers of the original instrument [46].

##### Expert Appraisal of Relevance

An expert appraisal of item relevance will be conducted at the end of step 4 by a 5-member subset of the committee (the principal researcher and 4 occupational health professionals who are familiar with the construct and content areas of the instrument) as part of a content validity assessment. This subset was selected for its content expertise, aligning with recommendations that compact panels of 5 to 10 experts enhance consistency and objectivity in content validity assessments [46,52]. This appraisal at step 4 is consistent with the guidance of Beaton et al [45] that substantive content decisions should precede pretesting while adding a quantitative check of cultural pertinence (content validity indexes; CVIs) to reinforce the prefinal version [46]. The objective is for the instrument to proceed to pretesting being conceptually stable and culturally contextualized, thereby reducing rework and preventing repeated translation–back translation cycles caused by low‑relevance items detected too late.

The experts will independently rate each item’s relevance to the target context using a 4‑point Likert‑type scale (1=“not at all relevant,” 2=“somewhat relevant,” 3=“quite relevant,” and 4=“highly relevant”). The item‑level CVI (I‑CVI) and the scale‑level CVI (S‑CVI; S-CVI/average method) will then be calculated following established thresholds (eg, I‑CVI≥0.80; S‑CVI/average method≥0.90) to guide refinement. Items falling below these cutoffs will be reworded without altering the underlying construct and subsequently returned to the committee for documented consensus [52]. Any partial or total item substitutions will require approval from the instrument developers prior to advancing in the adaptation process [46].

This appraisal will be conducted individually via an online form immediately following the meeting, and panelists will be allotted approximately 10 minutes to complete it and return their responses to the principal researcher. All resulting decisions (retaining, revising, replacing, or removing) and their justifications will be reviewed by the research team and finalized before proceeding to pretesting with the target population. The relevance appraisal will ensure that items entering pretesting are pertinent to the constructs in the Brazilian context, whereas the target population in the next step will verify how these items are understood and whether wording refinements are needed [45,46].

#### Step 5: Pretesting With the Target Population

Pretesting will involve administering the adapted prefinal version of the instrument to a recommended sample of 30 participants recruited from the target population [44,45,48,53]. The prefinal Brazilian Portuguese version will retain the original 23-item structure, 6 dimensions, and response format [33]. This step aims to assess another dimension of content validity, which is clarity. Clarity of the instructions, each item, and the response format of the questionnaire will be assessed [44,46].

Recruitment will prioritize heterogeneity in age, gender, and occupational sector [54] and will be conducted through the research team’s established communication network. Recruitment strategies will include email invitations, digital flyers, and dissemination via the team’s social media channels. Inclusion criteria are (1) Brazilian individuals aged 50 years and older, (2) current work full time or part time in Brazil in a formal or informal occupation, (3) at least 1 year in their current occupation, and (4) ability to read and understand Brazilian Portuguese. Exclusion criteria are individuals unable to provide informed consent or participate in an online interview. Before completing the questionnaire, participants must complete a brief complementary online form to provide socioeconomic, work-related, and health information (eg, age, gender, educational level, marital status, household composition, income, employment type, work schedule, sector, organization size, occupational exposures, chronic conditions, habits, disabilities, and self-rated well-being). This information will be provided remotely to the principal researcher.

After participants complete the online questionnaire under the guidance of the principal researcher, a cognitive debriefing interview will be conducted remotely and synchronously using videoconferencing platforms by the principal researcher. Participants will rate item clarity on a dichotomous scale (“it is clear” or “it is not clear”) and will suggest wording improvements for any item marked as “it is not clear” [44]. For each item, the proportion of participants rating it as “it is clear” will be calculated. Items identified as “it is not clear” by 20% or more of the participants will be flagged for revision to ensure comprehensibility for the target population. Interrater agreement will be considered acceptable when at least 80% of participants judge the item as clear [44,55]. Qualitative feedback provided during interviews will be synthesized through thematic analysis [56] to identify patterns and suggestions for improving clarity and wording. Items that generate comprehension problems will be revised and documented, and the original instrument developers will be consulted for any substantial changes [46].

The final wording of the prefinal Brazilian Portuguese version will be determined through a consensus-based process that integrates expert relevance ratings and committee recommendations from step 4 with participant feedback regarding item clarity and comprehensibility obtained during step 5.

#### Step 6: Field-Testing and Psychometric Evaluation

##### Overview

This step will integrate the field-testing of the prefinal version of the instrument with its psychometric validation, ensuring operational equivalence, high‑quality data, and evidence of measurement properties. All decisions, procedures, and document versions will be comprehensively documented to maintain transparency and traceability throughout the validation process [46,57-59].

The Brazilian Portuguese version of the EFCPT will retain the original structure, comprising 23 items distributed across 6 dimensions and assessed using a 5-point Likert response scale, including a “not applicable” response option. Dimension scores and an overall score will be calculated according to the original instrument guidelines [33], with higher scores indicating more frequent engagement in preventive work behaviors.

##### Target Population

The target population, as well as the recruitment procedures and eligibility criteria, will remain largely consistent with those established in step 5, ensuring methodological continuity across study phases. The main exceptions concern the minimum occupational tenure and the mode of data collection. For step 6, the minimum tenure in the current occupation will be reduced from 1 year to 6 months to increase participation while ensuring sufficient exposure to the occupational context and preventive work practices [60]. Additionally, participation will not depend on the ability to complete an online interview as face-to-face administration will be prioritized [61].

##### Sample Size

In the field of psychometric evaluation, there is no universal formula for defining sample size as it interacts with factors such as the number of items per factor, item communalities, type of correlation matrix (eg, Pearson or polychoric), and sample homogeneity [62-65]. Nevertheless, some authors still recommend simple ratios, such as a minimum of 5 participants per item [66-68]. In this study, we will adopt the recommendation of the COSMIN (Consensus-Based Standards for the Selection of Health Measurement Instruments) Study Design checklist [57,59], which advises recruiting 7 participants per item and at least 100 cases for studies assessing structural validity. Given that the EFCPT comprises 23 items, the 7:1 participant-to-item ratio results in a minimum sample size of 161 participants. This sample size is considered adequate for an initial assessment of the instrument’s dimensional structure and is consistent with recommendations for psychometric studies of structural validity [57,59]. Although larger samples are generally preferable for factor analytic procedures, the planned sample aligns with international standards [57,59,66,67] and represents a reasonable balance between methodological rigor and practical feasibility within the context of the present validation study.

##### Administration

Participants will complete the prefinal version of the instrument and a brief complementary questionnaire capturing sociodemographic, occupational, and health‑related characteristics to support hypothesis testing and score interpretability. Data collection will be conducted primarily through face-to-face sessions guided by the principal researcher or trained research team members. Field visits may be carried out when necessary to facilitate participation and reduce barriers related to internet access, digital literacy, or other logistical constraints. Remote synchronous administration may be used when face-to-face data collection is not feasible or when preferred by the participant. In such cases, flexible scheduling will be offered, including the possibility of completing the assessment across 2 sessions when necessary. Standardized administration procedures (eg, instructions, format, layout, sequencing of items, and training of data collectors) will be maintained across administration modes to uphold operational equivalence among respondents.

##### Data Quality Assessment and Preparation

Before modeling, data will be screened for missingness patterns and response distribution issues. Interpretability indicators will be examined, including score distributions and potential floor or ceiling effects. When pertinent, standard measurement quality indicators (eg, standard error of measurement; SE of measurement) will be estimated to complement reliability evidence and support interpretation of scores in the target population [46]. Sample adequacy will be assessed using the Kaiser-Meyer-Olkin measure and Bartlett test of sphericity, with Kaiser-Meyer-Olkin values of 0.80 or higher and a significant Bartlett test (*P*<.05) indicating suitability for factor analysis. These procedures ensure that the sample is adequate and that the data quality supports subsequent psychometric analyses.

##### Psychometric Evaluation

This step comprises the evaluation of the measurement properties of the Brazilian Portuguese version of the EFCPT focusing on structural validity and reliability [46,57,59].

Descriptive statistics, reliability analyses, and exploratory factor analysis (EFA) will be conducted using version 31.0 of the SPSS software (IBM Corp). The Amos software designed for SPSS (IBM Corp) will be used for confirmatory factor analyses (CFAs).

Missing data will be assessed regarding their frequency and pattern of occurrence. Participants with substantial missing responses will be excluded from the relevant psychometric analyses. For cases with a limited amount of missing data, appropriate statistical procedures will be applied according to the extent and nature of missingness.

##### Structural Validity

An EFA will be conducted using principal axis factoring, an extraction method appropriate for latent constructs, with an oblique rotation (Promax) given the theoretical expectation of correlated dimensions [46,69]. Factor retention will be guided by multiple criteria, including eigenvalues above 1.0, inspection of the scree plot, and salient item loadings (≥0.30) [62].

A CFA will subsequently be performed to provide a preliminary evaluation of the fit of the proposed factor structure. Model fit will be evaluated using the chi-square–to-df ratio, the comparative fit index, the goodness-of-fit index, and the root mean squared error of approximation. Following recommended criteria, comparative fit index and goodness-of-fit index values of 0.90 or higher and root mean squared error of approximation values of 0.08 or lower will be interpreted as indicative of acceptable fit; model refinements will be considered only when theoretically justified and supported by modification indexes [70,71].

Given the estimated sample size (N=161), the EFA and CFA will be conducted on the same full sample as splitting the dataset would result in underpowered subsamples. This approach is acceptable when sample size constraints prevent an adequately powered split, and the CFA will be interpreted conservatively as preliminary evidence of structural validity, with model modifications restricted to theoretically justified adjustments [72].

##### Reliability

Internal consistency will be evaluated for each dimension and for the total score using complementary coefficients (eg, Cronbach α and McDonald ω). Values of α of 0.70 or higher will be interpreted as acceptable, whereas α values of 0.80 or higher will indicate good reliability [46,73]. For test-retest reliability, an ICC will be calculated using a subsample of 32 participants recruited from the field-testing sample. Efforts will be made to ensure that the subsample reflects the demographic and occupational characteristics of the full validation sample. Participants will complete the instrument twice with a 2-week interval between administrations, minimizing recall bias while preserving stability [71]. An ICC of 0.75 or higher will be interpreted as good, and an ICC of 0.90 or higher will be interpreted as excellent; measurement error parameters may be derived to contextualize reliability results [74].

### Ethical Considerations

This study will adhere to all applicable ethical principles and has received ethics approval from the Research Ethics Committee of the University of Quebec in Trois‑Rivières (UQTR; CER-26-328-07.25) and from the Research Ethics Committee of the Federal University of Pernambuco (approval 8.260.740; CAAE 95375426.8.0000.5208).

## Results

The study received funding from the Mitacs Globalink Research Award in 2025 to 2026 to support activities related to the cross-cultural adaptation process and the principal investigator’s research internship at UQTR, Canada. From October 2025 to December 2025, a literature review was conducted, and the research protocol was developed.

From November 2025 to May 2026, the principal investigator completed a research internship at UQTR. This period supported the translation and cross-cultural adaptation process through methodological and linguistic input from French-speaking experts and collaboration with researchers affiliated with the center involved in the development of the original instrument.

Ethics applications were submitted to UQTR and the Federal University of Pernambuco in January 2026 and February 2026, respectively. Steps 1 (forward translation) and 2 (forward translation synthesis) were carried out in February 2026. Step 3 (back translation) was completed in March 2026, and step 4 (harmonization and expert appraisal of relevance) was conducted between March 2026 and April 2026.

Step 5 (pretesting with the target population) commenced in May 2026 and was concluded in August 2026. A total of 30 workers aged 50 years and older were enrolled and completed this phase. The timeline was slightly extended to facilitate participant recruitment and accommodate scheduling requirements. Step 6 is scheduled to begin in September 2026. Data analysis is expected to be completed by the end of February 2027, and the study findings are anticipated to be published in 2027.

## Discussion

### Expected Findings and Implications

This paper describes the protocol for the cross-cultural adaptation and psychometric validation of the Brazilian Portuguese version of the EFCPT among workers aged 50 years and older. The adaptation process is expected to result in an instrument with cultural relevance and conceptual equivalence that preserves the conceptual foundations of the original scale while ensuring its applicability within the Brazilian context. The study will also provide evidence regarding the structural validity and reliability of the Brazilian Portuguese version, supporting its use in occupational health research and practice.

Given the global aging of the workforce and the need to promote healthy and sustainable working lives, a validated instrument for assessing preventive work behaviors may facilitate the identification of factors associated with safer and healthier work environments for older workers in Brazil while enabling cross-cultural comparisons across countries [1,54,75]. These efforts are aligned with the World Health Organization’s Decade of Healthy Ageing (2021-2030), which recognizes the creation of age-friendly environments that support functional ability, participation, and well-being throughout the life course as a global priority [76].

Beyond the adaptation of the instrument itself, this protocol may offer methodological insights for future cross-cultural adaptation and validation studies involving older workers. By documenting the procedures, challenges, and recruitment strategies adopted throughout the study, particularly those designed to address barriers related to digital access and literacy, this research may help inform similar studies with aging worker populations in diverse occupational contexts.

A major strength of this study is the use of a rigorous and internationally recognized cross-cultural adaptation process involving forward translation, forward translation synthesis, back translation, harmonization and expert appraisal of relevance, pretesting with the target population, and field-testing and psychometric evaluation [44-46]. In addition, the research internship at UQTR facilitated collaboration with French-speaking experts and researchers involved in the development of the original instrument [33], supporting the linguistic and conceptual adaptation process. The planned psychometric evaluation will further provide evidence regarding the measurement properties of the Brazilian Portuguese version.

Potential limitations include challenges in recruiting adults aged 50 years and older and the possibility of selection bias associated with workplace-based recruitment. Experiences from the pretesting phase suggested that reliance on remote data collection could exclude individuals with limited internet access or lower digital literacy, a challenge that has been widely documented among older adult populations [61,77]. Therefore, the field phase will prioritize face-to-face administration and workplace visits to promote participation and reduce digital access barriers. In addition, the minimum occupational tenure required for participation in the field-testing phase was reduced from 1 year to 6 months to broaden eligibility while ensuring sufficient exposure to the occupational context and preventive work practices [60]. Despite efforts to maximize participation and diversity, the study sample may not fully represent the diversity of occupational sectors, employment contexts, and geographic regions across Brazil. These factors will be considered when interpreting and generalizing the study findings.

Workers aged 50 years and older were selected as the target population because this study is part of a broader research initiative focused on understanding and promoting healthy and sustainable work participation among aging workers. Consequently, the evidence generated will be most directly applicable to this population. Although the instrument may have potential applicability to the broader workforce [33,54], additional studies will be needed to establish its measurement properties in other age groups within the Brazilian workforce. Furthermore, while the proposed sample size is consistent with established psychometric recommendations, the interpretation of the psychometric findings should also consider the multidimensional structure of the instrument and the characteristics of the study sample.

Recognizing the potential contribution of the study findings to occupational health, healthy aging, and sustainable work participation, stakeholders engaged throughout the study will support the timely dissemination of preliminary findings during the data collection and analysis phases. Following completion of the study, a structured set of knowledge transfer activities will be implemented, including publication in peer-reviewed journals and presentations at national and international scientific conferences. Additional dissemination strategies may include workshops for occupational health and safety professionals, meetings with organizational stakeholders, and public outreach initiatives aimed at promoting the translation of research findings into practice.

Future research may expand validation efforts across diverse occupational sectors and regions of Brazil, examine the responsiveness of the instrument in longitudinal studies, and investigate associations between preventive behaviors and occupational health outcomes. Additional studies may also evaluate the feasibility and measurement equivalence of digital administration formats, assess the instrument in younger worker populations, and explore its integration into occupational health and safety monitoring and intervention programs.

### Conclusions

This paper describes a rigorous and internationally recognized process for the cross-cultural adaptation of what will be the first Brazilian Portuguese version of the EFCPT for use among workers aged 50 years and older, a population of increasing relevance in contemporary labor markets. The adapted instrument is expected to support cross-national research; inform evidence-based workplace interventions; and contribute to the promotion of safe, inclusive, and sustainable work participation among aging workers. Moreover, the detailed description of the study procedures may serve as a methodological reference for researchers planning and conducting similar cross-cultural adaptation and psychometric validation studies.

## Abbreviations

CFA: confirmatory factor analysis
COSMIN: Consensus-Based Standards for the Selection of Health Measurement Instruments
CVI: content validity index
EFA: exploratory factor analysis
EFCPT: Preventive Behaviors at Work Frequency Scale
I-CVI: item-level content validity index
ICC: intraclass correlation coefficient
MPBW: model of preventive behaviors at work
S-CVI: scale-level content validity index
UQTR: University of Quebec in Trois-Rivières

## Data Availability

Data sharing is restricted due to ethical considerations and the inclusion of personal and occupational information. Deidentified data generated during this study may be made available from the corresponding author on reasonable request and subject to approval by a research ethics committee in accordance with the approved ethics protocol.

## References

[1] dos Santos MM, da Silva WM, de Moura Pereira A, de Carvalho WS (2025). Active aging and the inclusion of older adults in the labor market: a narrative review [Article in Portuguese]. Rev Foco.

[2] (2025). Envelhecimento da população impulsiona novas ações em defesa dos idosos [Article in Portuguese]. Senado Federal.

[3] (2025). Ageing: global population. World Health Organization.

[4] Feijó J (2025). Geração prateada: onde estão os 60+ no mercado de trabalho [Article in Portuguese]. Brazilian Institute of Economics, Fundação Getulio Vargas.

[5] (2024). Relação anual de informações sociais- RAIS [Article in Portuguese]. Ministério do Trabalho e Emprego.

[6] Pazos PF, Bonfatti RJ (2020). Velhice, trabalho e saúde do trabalhador no Brasil: uma revisão integrativa [Article in Portuguese]. Rev Bras Geriatr Gerontol.

[7] Ribeiro PC, Almada DS, Souto JF, Lourenço RA (2018). Permanence in the labour market and life satisfaction in old age [Article in Portuguese, English]. Cien Saude Colet.

[8] Silva-Junior JS, Martinez MC, Sekiya FS, de Miranda CB, Fischer FM (2023). Return to work after sick leave due to musculoskeletal disorder or injury: a longitudinal study conducted in Brazil. BMC Public Health.

[9] Cancelliere C, Donovan J, Stochkendahl MJ, Biscardi M, Ammendolia C, Myburgh C, Cassidy JD (2016). Factors affecting return to work after injury or illness: best evidence synthesis of systematic reviews. Chiropr Man Therap.

[10] (2021). Quantidade de acidentes do trabalho, por situação do registro e motivo, segundo os grupos de idade e sexo, no Brasil - 2022/2024 [Article in Portuguese]. Ministério da Previdência Social.

[11] Lancman S, Barroso BI (2021). Mental health: professional rehabilitation and the return to work - a systematic review. Work.

[12] (2024). Costs of workplace accidents and illnesses worldwide. International Labour Organization.

[13] Chen H, Richard OC, Dorian Boncoeur O, Ford DL Jr (2020). Work engagement, emotional exhaustion, and counterproductive work behavior. J Bus Res.

[14] Demerouti E, Bakker AB, Halbesleben JR (2015). Productive and counterproductive job crafting: a daily diary study. J Occup Health Psychol.

[15] Smartlab.

[16] (2025). “Prevenção é essencial para um ambiente de trabalho seguro”, destaca Luiz Marinho na Canpat 2025 [Article in Portuguese]. Ministério do Trabalho e Emprego.

[17] Loisel P, Buchbinder R, Hazard R, Keller R, Scheel I, van Tulder M, Webster B (2005). Prevention of work disability due to musculoskeletal disorders: the challenge of implementing evidence. J Occup Rehabil.

[18] Loisel P, Durand MJ, Berthelette D, Vézina N, Baril R, Gagnon D, Larivière C, Tremblay C (2001). Disability prevention: new paradigm for the management of occupational back pain. Dis Manag Health Outcomes.

[19] Hurtado SL, Silva-Macaia AA, Vilela RA, Querol MA, Lopes MG, Bezerra JL (2022). Occupational health interventions - context, challenges and possibilities for development: a scoping review [Article in Portuguese]. Rev Bras Saude Ocup.

[20] Lecours A (2019). Using an occupational perspective to understand behaviours fostering the prevention of work-related health problems: a proposed conceptual model. J Occup Sci.

[21] Casey T, Griffin MA, Flatau Harrison H, Neal A (2017). Safety climate and culture: integrating psychological and systems perspectives. J Occup Health Psychol.

[22] Griffin MA, Neal A (2000). Perceptions of safety at work: a framework for linking safety climate to safety performance, knowledge, and motivation. J Occup Health Psychol.

[23] DeJoy DM, Wilson MG, Vandenberg RJ, McGrath‐Higgins AL, Griffin‐Blake CS (2010). Assessing the impact of healthy work organization intervention. J Occup Organ Psychol.

[24] DeJoy DM, Wilson MG (2003). Organizational health promotion: broadening the horizon of workplace health promotion. Am J Health Promot.

[25] de Miranda Prottes V, Oliveira NC, de Oliveira Andrade AB (2012). Ergonomic work analysis as a tool of prevention for the occupational safety and health management system. Work.

[26] Lecours A (2020). Scientific, professional and experiential validation of the model of preventive behaviours at work: protocol of a modified Delphi study. BMJ Open.

[27] Lecours A (2025). Le modèle des comportements préventifs au travail: une ressource validée par la recherche pour optimiser les sains comportements des travailleurs et la santé des organisations [Article in French]. Can J Behav Sci.

[28] Lecours A, Therriault PY (2016). French transcultural validation of the Compliance with Safety Behavior Scale. Work.

[29] Lecours A, Therriault PY (2017). Preventive behaviour at work - a concept analysis. Scand J Occup Ther.

[30] Lecours A, Therriault PY (2018). Development of preventive behavior at work: description of occupational therapists' practice. Work.

[31] Lecours A, St-Hilaire F, Daneau P (2021). Moving toward an integrated prevention approach for mental health at work: promoting workers' involvement through concrete actions. Work.

[32] Lecours A, Therriault PY (2019). Habiliter les travailleurs la prévention: description des pratiques des ergothérapeutes visant la mise en place des antécédents du comportement préventif au travail [Article in French]. Rev Francoph Rech Ergother.

[33] Lecours A, Beaulieu AA, Poulin V, Nastasia I, St-Hilaire F (2023). Échelle de fréquence des comportements préventifs au travail: analyse des propriétés métrologiques [Article in French]. Can J Behav Sci.

[34] de Mello Alves MG, Chor D, Faerstein E, de S Lopes C, Werneck GL (2004). Short version of the "Job Stress Scale": a Portuguese-language adaptation [Article in Portuguese]. Rev Saude Publica.

[35] Lima IA, Parma GO, Cotrim TM, Moro AR (2019). Psychometric properties of a medium version of the Copenhagen Psychosocial Questionnaire (COPSOQ II) for southern Brazil. Work.

[36] Silva MA, Wendt GW, Argimon II (2017). Propriedades psicométricas das medidas do Questionário Psicossocial de Copenhague I (COPSOQ I), versão curta [Article in Portuguese]. REGE Rev Gest.

[37] Gonçalves JS, Moriguchi CS, Chaves TC, de Oliveira Sato T (2021). Cross-cultural adaptation and psychometric properties of the short version of COPSOQ II-Brazil. Rev Saude Publica.

[38] Melo JS, Dibai-Filho AV, Rêgo AS, Rocha DS, de Walsh IA, de Jesus Tavarez RR, Gonçalves MC, Damasceno KL, de Paula Gomes CA, Bassi-Dibai D (2022). Translation, cross-cultural adaptation and validation of the Work Role Functioning Questionnaire 2.0 into Brazilian Portuguese in a general population. Health Qual Life Outcomes.

[39] Pinto Melo JS, Dibai-Filho AV, Brito Pinheiro CA, Rêgo AS, Bezerrade Castro Soares KV, Brito Damasceno KL, Pontes-Silva A, Fidelis de Paula Gomes CA, Arias Avila M, Bassi-Dibai D (2024). The Brazilian version of the Work Role Functioning Questionnaire 2.0 with 5 items (WRFQ-5) has adequate measurement properties. Physiother Theory Pract.

[40] Greco PB, Munhoz OL, da Luz EM, Seidel Junior E, McEwen K, Magnago TS (2024). Tradução e adaptação transcultural da Resilience at Work Scale para o Português do Brasil [Article in Portuguese]. Texto Contexto Enferm.

[41] Gasparino RC, da Silva SM, de Barros Minatogawa LB, Ribeiro OM, Bernardes A (2024). Cross-cultural adaptation and validation of the Healthy Work Environment Assessment Tool in Brazilian culture [Article in English, Portuguese]. Rev Bras Enferm.

[42] Silva-Junior JS, Ogata AJ, Roviello VD, Barcelos RC, Pedro TB, Gallasch CH (2026). Cross-cultural adaptation and content validity of the 2019 CDC Worksite Health ScoreCard for use in Brazil. Am J Health Promot.

[43] Brinkhof LP, Ridderinkhof KR, Murre JM, Krugers HJ, de Wit S (2025). Boosting behavioral adaptability to enhance older adults' mental health/well-being and quality of life using a habit-based metacognitive self-help intervention. BMC Psychol.

[44] Sousa VD, Rojjanasrirat W (2011). Translation, adaptation and validation of instruments or scales for use in cross-cultural health care research: a clear and user-friendly guideline. J Eval Clin Pract.

[45] Beaton DE, Bombardier C, Guillemin F, Ferraz MB (2000). Guidelines for the process of cross-cultural adaptation of self-report measures. Spine (Phila Pa 1976).

[46] Cruchinho P, López-Franco MD, Capelas ML, Almeida S, Bennett PM, Miranda da Silva M, Teixeira G, Nunes E, Lucas P, Gaspar F (2024). Translation, cross-cultural adaptation, and validation of measurement instruments: a practical guideline for novice researchers. J Multidiscip Healthc.

[47] Coster WJ, Mancini MC (2015). Recommendations for translation and cross-cultural adaptation of instruments for occupational therapy research and practice. Rev Ter Ocup Univ São Paulo.

[48] Beaton D, Bombardier C, Guillemin F, Ferraz MB (2007). Recommendations for the cross-cultural adaptation of the DASH and QuickDASH outcome measures. Institute for Work & Health.

[49] Jones EG, Kay M (1992). Instrumentation in cross-cultural research. Nurs Res.

[50] van de Vijver F, Tanzer NK (2004). Bias and equivalence in cross-cultural assessment: an overview. Eur Rev Appl Psychol.

[51] Hedrih V (2019). Adapting Psychological Tests and Measurement Instruments for Cross-Cultural Research: An Introduction.

[52] Almanasreh E, Moles R, Chen TF (2019). Evaluation of methods used for estimating content validity. Res Social Adm Pharm.

[53] Perneger TV, Courvoisier DS, Hudelson PM, Gayet-Ageron A (2015). Sample size for pre-tests of questionnaires. Qual Life Res.

[54] Lecours A, Beaulieu AA, Poulin V, Nastasia I, St-Hilaire F (2021). Development of a questionnaire measuring preventive behaviors at work. J Occup Rehabil.

[55] Topf M (1986). Three estimates of interrater reliability for nominal data. Nurs Res.

[56] Braun V, Clarke V (2008). Using thematic analysis in psychology. Qual Res Psychol.

[57] Mokkink LB, Prinsen CA, Patrick DL, Alonso J, Bouter LM, de Vet HC, Terwee CB (2019). COSMIN study design checklist for patient-reported outcome measurement instruments. COSMIN.

[58] Field A (2018). Why is my evil lecturer forcing me to learn statistics?. Discovering Statistics Using IBM SPSS Statistics.

[59] Prinsen CA, Mokkink LB, Bouter LM, Alonso J, Patrick DL, de Vet HC, Terwee CB (2018). COSMIN guideline for systematic reviews of patient-reported outcome measures. Qual Life Res.

[60] Silva AR, Gomes CA, Silva Júnior JE, Rocha DS, Pinheiro CA, Dibai Filho AV, Bassi-Dibai D (2021). Translation, cross-cultural adaptation, and reliability of the Workplace Sitting Breaks Questionnaire into Brazilian Portuguese. Rev Assoc Med Bras (1992).

[61] Khan S, Webster S, Puxty J, Robertson M (2026). Key challenges and barriers to digital literacy for older adults: scoping review. JMIR Aging.

[62] Lloret-Segura S, Ferreres-Traver A, Hernández-Baeza A, Tomás-Marco I (2014). Exploratory item factor analysis: a practical guide revised and up-dated [Article in Spanish]. Ann Psychol.

[63] Ferrando PJ, Anguiano-Carrasco C (2010). El análisis factorial como técnica de investigación en psicología [Article in Spanish]. Papeles del Psicólogo.

[64] Fabrigar LR, Wegener DT, MacCallum RC, Strahan EJ (1999). Evaluating the use of exploratory factor analysis in psychological research. Psychol Methods.

[65] Beavers AS, Lounsbury JW, Richards JK, Huck SW, Skolits GJ, Esquivel SL (2013). Practical considerations for using exploratory factor analysis in educational research. Pract Assess Res Eval.

[66] Norman GR, Streiner DL (2008). Biostatistics: The Bare Essentials.

[67] Howard MC (2016). A review of exploratory factor analysis decisions and overview of current practices: what we are doing and how can we improve?. Int J Hum Comput Interact.

[68] Gorsuch RL (1983). Factor Analysis.

[69] Haig BD (2005). Exploratory factor analysis, theory generation, and scientific method. Multivariate Behav Res.

[70] Marôco J (2021). Etapas da análise de equações estruturais. Análise de Equações Estruturais, Fundamentos Teóricos, Software e Aplicações [Book in Portuguese].

[71] Streiner DL, Norman GR, Cairney J (2024). Health Measurement Scales: A Practical Guide to Their Development and Use.

[72] Kyriazos TA (2018). Applied psychometrics: sample size and sample power considerations in factor analysis (EFA, CFA) and SEM in general. Psychology.

[73] Gray JR, Grove SK, Sutherland S (2021). Burns & Grove's the Practice of Nursing Research: Appraisal, Synthesis, and Generation of Evidence.

[74] Koo TK, Li MY (2016). A guideline of selecting and reporting intraclass correlation coefficients for reliability research. J Chiropr Med.

[75] (2019). Working better with age. Organisation for Economic Co-operation and Development.

[76] (2020). Decade of healthy ageing: baseline report. World Health Organization.

[77] Oh SS, Kim KA, Kim M, Oh J, Chu SH, Choi J (2021). Measurement of digital literacy among older adults: systematic review. J Med Internet Res.

